# Causal association between air pollution and frailty: a Mendelian randomization study

**DOI:** 10.3389/fpubh.2023.1288293

**Published:** 2023-11-07

**Authors:** Haixia Xiao, Shan Huang, Wei Yang, Wenni Zhang, Huanshun Xiao, Shuangming Cai

**Affiliations:** ^1^Department of Obstetrics, Guangdong Women and Children Hospital, Guangzhou, China; ^2^Department of MICU, Guangdong Women and Children Hospital, Guangzhou, China; ^3^Department of Internal Medicine, Guangdong Women and Children Hospital, Guangzhou, China

**Keywords:** air pollution, PM_2.5_, frailty, causal association, GWAS, Mendelian randomization

## Abstract

**Backgrounds:**

Frailty is a significant problem for older persons since it is linked to a number of unfavorable consequences. According to observational researches, air pollution may raise the risk of frailty. We investigated the causal association between frailty and air pollution (including PM_2.5_, PM_2.5–10_, PM_10_, nitrogen dioxide, and nitrogen oxides) using Mendelian randomization approach.

**Methods:**

We conducted MR analysis using extensive publically accessible GWAS (genome-wide association studies) summary data. The inverse variance weighted (IVW) method was employed as the primary analysis method. The weighted median model, MR-Egger, simple model, and weighted model approaches were chosen for quality control. The Cochran’s Q test was utilized to evaluate heterogeneity. Pleiotropy is found using the MR-Egger regression test. The MR-PRESSO method was used to recognize outliers. The leave-one-out strategy was used to conduct the sensitivity analysis.

**Results:**

MR results suggested that PM_2.5_ was statistically significantly associated with frailty [odds ratio (OR) = 1.33; 95%confidence interval (CI) = 1.12–1.58, *p* = 0.001] in IVW method. We observed no statistical association between PM_2.5–10_(OR = 1.00, 95% CI = 0.79–1.28, *p* = 0.979), PM_10_(OR = 0.91, 95% CI = 0.75–1.11, *p* = 0.364), nitrogen dioxide (OR = 0.98, 95% CI = 0.85–1.12, *p* = 0.730), nitrogen oxides (OR = 1.15, 95% CI = 0.98–1.36, *p* = 0.086) and frailty. There was no pleiotropy in the results. The sensitivity analysis based on the leave-one-out method showed that the individual single nucleotide polymorphisms (SNPs) did not affect the robustness of the results.

**Conclusion:**

The current MR investigation shows a causal association between PM_2.5_ and frailty. Frailty’s detrimental progression may be slowed down with the help of air pollution prevention and control.

## 1. Introduction

Frailty is a complicated age-related clinical syndrome marked by vulnerability, significant dysregulation in an aging body’s biologically complex dynamical system, and increased susceptibility to stress (1–3). Epidemiological evidence emphasizes the increasing frequency of frailty in older alduts as the population ages at an accelerated rate. For instance, a thorough meta-analysis of 57 research revealed that around 26.8% of the older population suffers from frailty (4). Frailty is not only a major risk factor for mortality in older adults, but is associated with a number of unfavorable events, such as falls (5), fractures (6), hospitalization (7), and reduced quality of life (8). Thus, frailty has become an important public health problem and a global health burden.

Because of the potential health consequences, air pollution is the leading reversible environmental factor linked to early mortality or disability (9). Particulate matter (PM) or gases like ozone, carbon monoxide, nitrogen dioxide (NO_2_), sulfur dioxide, and nitrogen oxides (NOx) are popular categories for air pollution (10). The most frequently studied pollutant is fine particulate matter with a diameter of less than 2.5 micrometers (PM_2.5_), followed by nitrogen dioxide (NO_2_) and nitrogen oxides (NOx), with few research concentrating on other pollutants. There is growing epidemiologic evidence that exposure to air pollution is associated with a variety of health problems, including respiratory disease (11), cardiovascular disease (12), cerebrovascular disease (13), mental health (14), and cancer (15, 16). The largest health issue is PM_2.5_ because it is likely that it contains a more hazardous mixture and because it enters the lungs more deeply than larger particles (17).

Some evidence from observational studies suggests a link between air pollution and frailty. A large cross-sectional study that included 220,079 subjects aged 60 years and older demonstrated that frailty and pre-frailty were substantially correlated with exposure to air pollutants such PM_2.5_, PM_2.5–10_, or NOx (18). In addition, a Korean study also showed that PM_2.5_ and PM_10_ exposures were commonly associated with pre-frailty and frailty among 2,912 community-dwelling individuals aged ≥ 70 years (19). More recently, results from a related study of middle-aged and older Chinese alduts also showed that Long-term exposure to PM was linked to a greater incidence of frailty deterioration (20). However, these observational studies may have methodological problems, such as homologous or reverse causation, leading to differences in results that may be due to residual confounders or reverse causation in observational studies. To address these concerns and establish a more robust causal relationship, randomized controlled trials (RCTs) stand as the preferred methodological approach, as they circumvent the confounding variables inherent to observational research. However, in the context of frailty and its association with air pollution, it is important to note that there is a notable absence of RCTs specifically designed to investigate this relationship.

Mendelian randomization (MR) is increasingly used in assessing whether correlations are consistent with causal hypotheses and can solve the confounding and reverse causality issues in observational studies. MR studies are instrumental variable analyses that use genetic variants (single nucleotide polymorphisms; SNPs) as exposed instrumental variables (21). MR studies are often described as “natural RCTs” because the random assignment of alleles during meiosis is conceptually similar to an RCT design. In this study, the MR approach makes use of genetic variants that exist in nature and which are considered to be randomly assigned. This means that there is no selective relationship between an individual carrying a certain genetic variant and exposure to air pollution, and therefore it can be considered as a natural random assignment. In addition, MR methods can reduce the possibility that confounding factors (e.g., age, gender, lifestyle, etc.) may interfere with the results of a study by analyzing the relationship between genetic variants and frailty. Furthermore, MR methods provide stronger causal inference. Therefore, the use of MR methods to study the association between air pollution and frailty can overcome some of the difficulties in traditional observational studies and provide more reliable causal evidence, which helps us to study the association between air pollution and frailty in greater depth while reducing the interference of confounding factors. As a result, in order to further investigate whether there is a connection between air pollution and frailty, we used extensive, publicly available genome-wide association study (GWAS) data with frailty as the endpoint and PM_2.5_, PM_2. 5–10_, PM_10_, nitrogen dioxide (NO_2_), and nitrogen oxides (NOx) as the exposure factors.

## 2. Materials and methods

### 2.1. Study design

**Figure 1:**
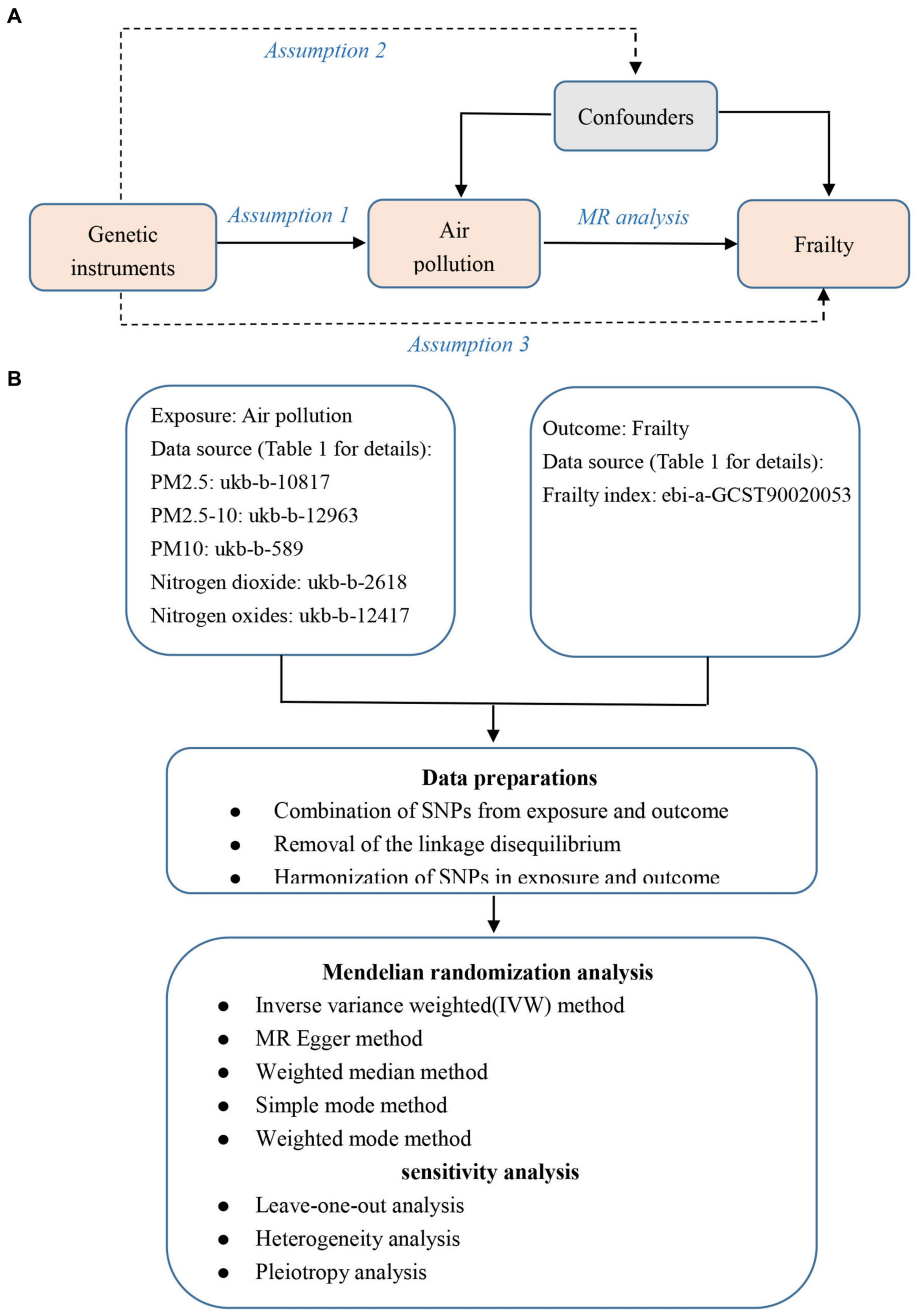
The overview flowchart of hypothesis and schematic design. **(A)** Mendelian randomization key hypothesis Diagram. SNPs associated with air pollution were used as the genetic instruments for investigating the causal effect of air pollution on frailty. Line with arrows indicates that the genetic instruments (SNPs) are associated with the exposure and can only affect the outcome via the exposure. Dashed lines indicate that the genetic instruments (SNPs) are independent of confounders between the results. **(B)** Schematic design for the Mendelian randomization analysis.

Our study is based on the Mendelian randomization design, which is predicated on three key tenets: (1) that instrumental variants are related to exposure; (2) that they are not related to outcome via a confounding pathway; and (3) that they do not directly affect outcome, only perhaps indirectly through exposure (Figure 1A). The exposure factor in this study was air pollution (PM_2.5_, PM_2.5–10_, PM_10_, nitrogen dioxide, and nitrogen oxides), the instrumental variables (IVs) were single nucleotide polymorphisms (SNPs) strongly related to air pollution, and the outcome variable was frailty. Here, we used a two-sample MR analysis to determine the causative relationships between frailty and air pollution. Figure 1 shows the flowchart for this Mendelian randomization investigation.

### 2.2. Data sources

Table 1 lists the data sources in detail. As exposures, we chose air pollution (including PM_2.5_, PM_2.5–10_, PM_10_, nitrogen dioxide, and nitrogen oxides), with data on all air pollution coming from UK Biobank, a sizable prospective study with more than 500,000 participants from the United Kingdom, and for which data on phenotypes, genetic information, and genome-wide genotyping have been published (22). Participants included in the GWAS summary datasets for PM_2.5_ (GWAS ID: ukb-b-10817), PM_2.5–10_ (GWAS ID: ukb-b-12963), PM_10_ (GWAS ID: ukb-b-589), nitrogen dioxide (GWAS ID: ukb-b-2618), and nitrogen oxides (GWAS ID: ukb-b-12417) were 423,796, 423,796, 455,314, 456,380, 456,380 participants, respectively. All participants had given informed consent in the corresponding original studies.

**Table 1:** Overview of data sources in this two-sample MR study.

Exposures/outcomes		Dataset	Sample size	Number of SNPs	Population	Consortium	Sex	Year
Particulate matter (PM)	PM_2.5_ um	ukb-b-10817	423,796	9,851,867	European	MRC-IEU	Males and females	2018
	PM_2.5–10_ um	ukb-b-12963	423,796	9,851,867	European	MRC-IEU	Males and females	2018
	PM_10_ um	ukb-b-589	455,314	9,851,867	European	MRC-IEU	Males and females	2018
Nitrogen dioxide		ukb-b-2618	456,380	9,851,867	European	MRC-IEU	Males and females	2018
Nitrogen oxides		ukb-b-12417	456,380	9,851,867	European	MRC-IEU	Males and females	2018
Frailty index		ebi-a-GCST90020053	175,226	7,589,717	European	NA	NA	2021

PM, Particulate matter; MR, Mendelian randomization; SNPs, Single nucleotide polymorphisms; MRC-IEU, Medical Research Council Integrative Epidemiology Unit; NA, Not available.

The most recent meta-GWAS study concentrating on the genetic architecture of frailty was used to generate summary statistics for the relationships of the instrumental factors with frailty (23). The participants in this study were 10,616 Swedish participants from the TwinGene, aged 41–87, and 164,610 European participants from the UK Biobank, aged 60–70. The frailty index (FI), which is based on the accumulation of a number of health deficiencies over the course of a person’s life, including symptoms, disabilities, and diagnosed diseases, was used in this meta-GWAS to characterize frailty. 49 and 44 self-reported baseline data variables from the UK Biobank and TwinGene, respectively, were used to derive the FI.

### 2.3. Selection of instrumental variables

As seen in Figure 1B, the genome-wide significance criterion for exposure was set at *p* < 5× 10^−8^ in order to satisfy assumption 1, however only PM_10_ (ukb-b-589) was able to identify enough SNPs (24). The results of previous study demonstrated the low potential of weak instrumental variable bias in MR analysis following the linear regression of each genetic variant on risk variables at *p* < 1 × 10^−6^ as a screening threshold (25). Therefore, we reduced the genome-wide significance threshold of the remaining exposure (ukb-b-10817, ukb-b-12963, ukb-b-2618, and ukb-b-12417) to *p* < 1× 10^−6^in order to pick sufficient SNPs as IVs associated with this significance level.

When extracting IVs, r^2^ < 0.001 and kb > 10,000 were selected to eliminate SNPs with linkage disequilibrium (LD) (26). The proxy SNP in linkage disequilibrium (r^2^ > 0.8) was utilized if the chosen SNP was not included in the resultant GWAS (27). To make sure that the effect of these SNPs on exposure related to the same allele as the effect on outcome, palindromic SNPs were then eliminated.

Finally, for each SNP, we determined the R^2^ (28) and F-statistic (29) as described previously. The F statistic is used to determine whether a weak IV bias is present. R^2^ is the proportion of iron status variability. Each SNP we chose had an F-statistic of greater than 10, indicating that the genetic tests we chose accurately predicted the exposure (30). The Supplementary Table provide specific SNP information together with the matching R^2^ and F-statistic.

### 2.4. Mendelian randomization analysis

Using the inverse variance weighted (IVW) approach, the causal connection between air pollution and frailty was evaluated. Through the Wald ratio, IVW can obtain an estimate of the causal effect based on a single genetic IV and then choose a fixed effect model to carry out a meta-analysis of various estimates of the causal effect based on a single gene IV. This method can produce a trustworthy estimate of the causal effect and is frequently used in MR Analysis (25). To improve accuracy and stability, we included further verification using MR-Egger regression, weighted median, weighted mode, and simple mode (31). To determine whether pleiotropy in IVs existed and whether it had an impact on the outcomes, we utilized MR-Egger regression. If the MR-Egger intercept was close to zero or the significance level was greater than 0.05, it was determined that pleiotropy had no impact on IVs (32). Cochran’s Q test was used to evaluate heterogeneity among IVs for the IVW approach (33). There was no heterogeneity, as evidenced by the finding of *p* > 0.05. The MR-PRESSO approach was additionally utilized to identify outliers (SNPs) and to offer a causal estimate when associated outliers are eliminated (34). We performed a leave-one-out sensitivity test, removing each SNP one at a time, to see if any particular SNP affected our results after removing random errors from screening IVs (35).

### 2.5. Statistical analysis

All analyses were conducted with the “TwoSampleMR” (26) package and “MR-PRESSO” (34) in R Foundation version 4.2.2. The threshold of statistical significance for evidence is *p* < 0.05.

## 3. Results

### 3.1. Genetic IVs extraction of air pollution from the frailty GWAS dataset

In the present study, after removing chained imbalanced IVs from the frailty GWAS dataset, we identified a number of independent SNPs linked to following exposures (*p* < 1 × 10^−6^): 21 strongly related SNPs to PM_2.5_, 6 strongly related SNPs to PM_2.5–10_, 34 strongly related SNPs to nitrogen dioxide, 30 strongly related SNPs to nitrogen oxides. We identified 19 independent SNPs with *p* < 5 × 10^−8^ linked to PM_10_. The effect allele frequency (EAF) of SNPs in PM_2.5_, PM_2.5–10_, PM_10_, nitrogen dioxide and nitrogen oxides ranged from 0.004 to 0.658, 0.020 to 0.257, 0.015 to 0.640, 0.004 to 0.623 and 0.004 to 0.962, respectively. In addition, to exclude the potential impacts of weak IVs, we used F statistic to test the correlation strength of IVs with exposure, and no evidence of significant weak IVs among the selected SNPs were observed (all *F* > 10), indicating that a weak instrumental bias was unlikely to affect the estimation of the causal effects. The Supplementary Table included information about these IVs in detail.

### 3.2. Mendelian randomization analysis

**Figure 2:**
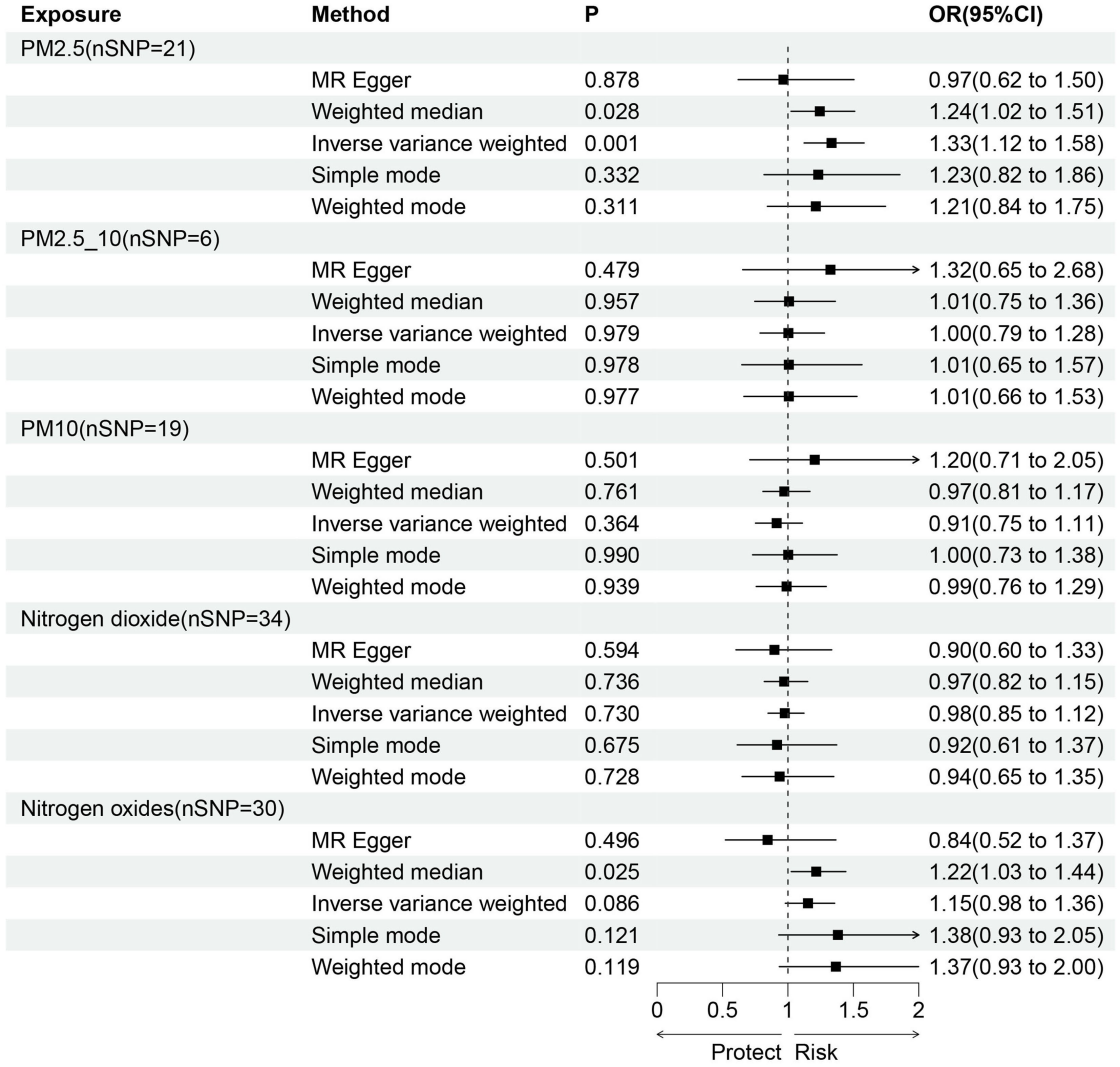
The causal relationship between exposure to air pollution and outcomes in frailty. nSNP, number of SNPs; OR, odds ratio; CI, confidence interval.

**Figure 3:**
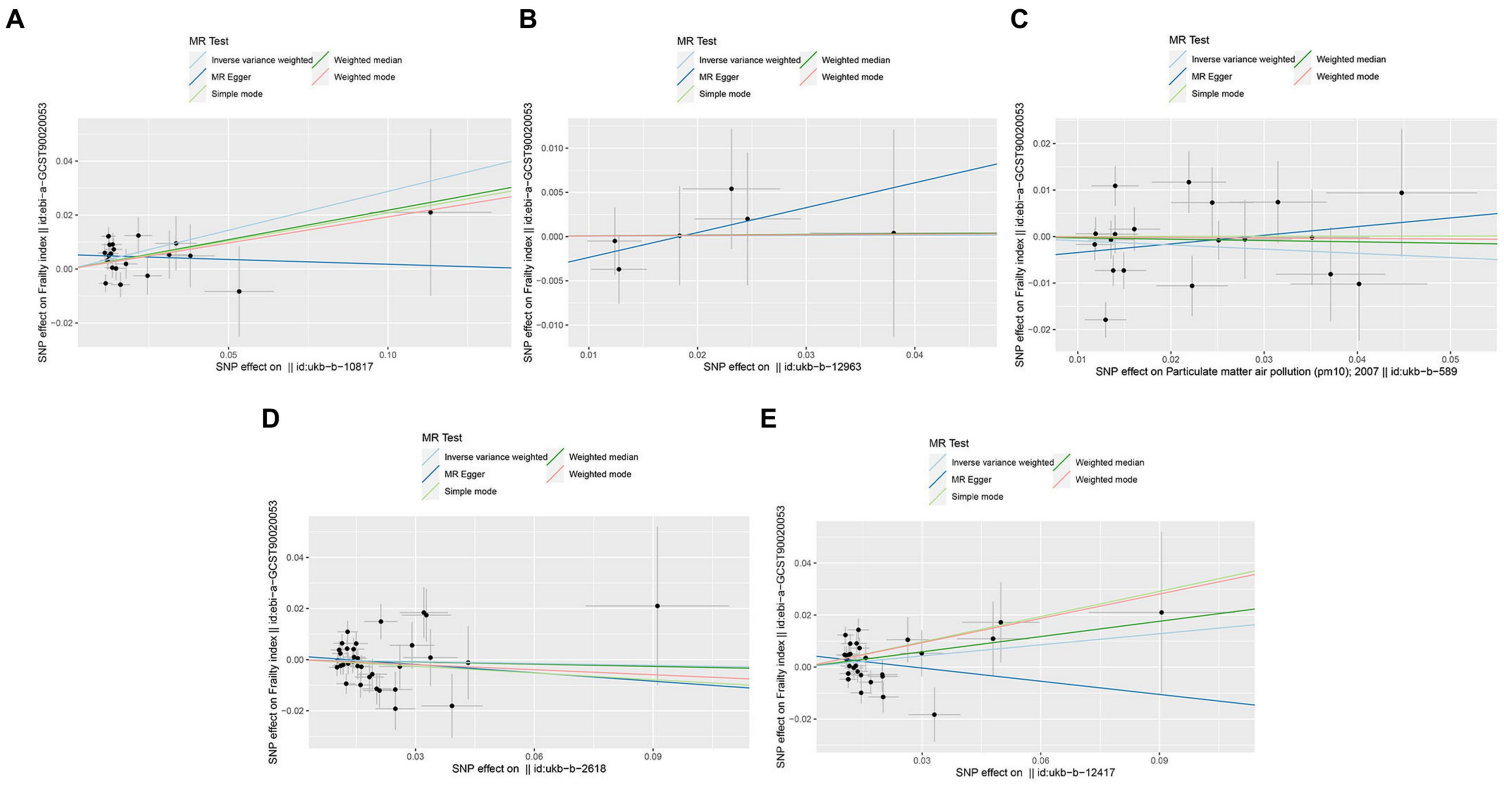
Scatter plots of SNPs associated with air pollution and frailty. Each black point representing each SNP on the exposure (horizontal-axis) and on the outcome (vertical-axis) is plotted with error bars corresponding to each standard error (SE). The Mendelian randomization (MR) regression slopes of the lines represent the causal estimates using five approaches (inverse-variance weighted (IVW), MR-Egger, weighted median, simple mode, and weighted mode). **(A)** PM_2.5_. **(B)** PM_2.5–10_. **(C)** PM_10_. **(D)** Nitrogen dioxide. **(E)** Nitrogen oxides.

**Figure 4:**
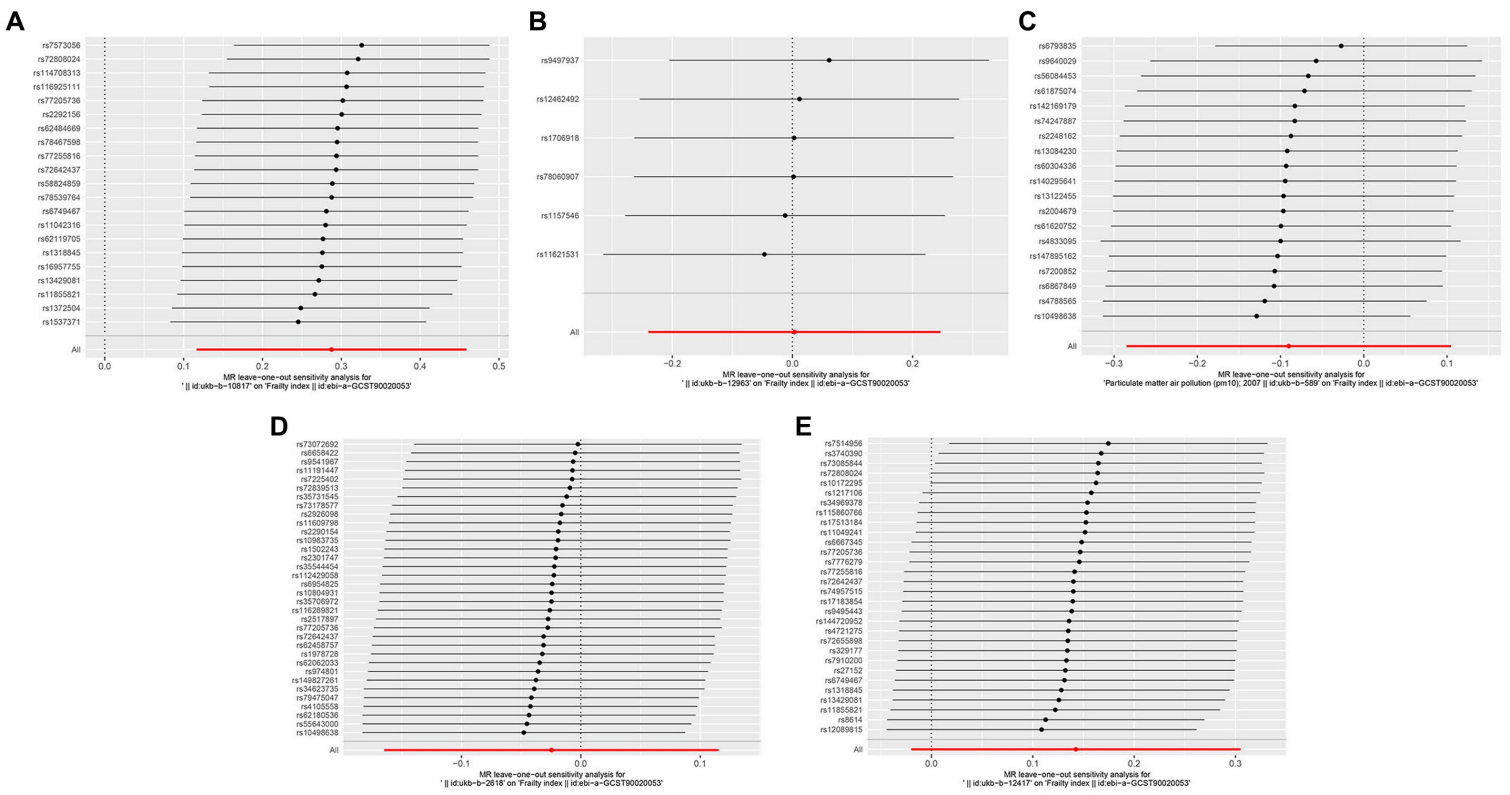
Forest plots of Leave-one-out analyses for causal SNP effect of air pollution on frailty. The error bars indicate the 95% confidence interval (CI). **(A)** PM_2.5_; **(B)** PM_2.5–10_; **(C)** PM_10_; **(D)** Nitrogen dioxide; **(E)** Nitrogen oxides.

In the primary IVW analyses, following are the odds ratios (ORs) and 95% confidence intervals (CIs) of various exposures that our MR study showed: PM2.5: OR = 1.33, 95% CI = 1.12–1.58, *p* = 0.001; PM2.5–10: OR = 1.00, 95% CI = 0.79–1.28, *p* = 0.979; PM10: OR = 0.91, 95% CI = 0.75–1.11, *p* = 0.364; nitrogen dioxide: OR = 0.98, 95% CI = 0.85–1.12, *p* = 0.730; nitrogen oxides: OR = 1.15, 95% CI = 0.98–1.36, *p* = 0.086 (Figure 2). As shown in the results, we discovered a causal association between PM_2.5_ and frailty. Besides, the weighted median method also revealed a connection between PM_2.5_ and the risk of frailty (OR = 1.24, 95% CI = 1.02–1.51, *p* = 0.028; Figure 2). In the MR-Egger, weighted model, and simple model, these relationships remained but were not statistically significant (Figure 2). These results showed that the risk of frailty arose considerably with each standard error increase in the level of PM_2.5_ exposure (Figures 2–4). Additionally, no evidence of causal relationships between PM_2.5–10_, PM_10_, nitrogen dioxide, nitrogen oxides and the risk of frailty was found in this study (Figures 2–4).

### 3.3. Pleiotropy and heterogeneity analysis

The results of the MR-Egger intercept test revealed no pleiotropy (*p* > 0.05; Table 2). This showed that, independent of the corresponding exposures, none of the SNPs identified from exposures contribute to frailty. Heterogeneity was observed in our study (PM_2.5_, *p* = 0.010, Q = 37.598; PM_10_, *p* < 0.001 Q = 44.526; Nitrogen dioxide, *p* = 0.004 Q = 58.330; Nitrogen dioxide, *p* < 0.001 Q = 64.132; Table 2).

**Table 2:** Pleiotropy and heterogeneity test of air pollution genetic instrumental variables in GWAS for frailty.

Exposures	Pleiotropy test			Heterogeneity test		
	P	SE	Intercept	P	Q	Q_df
PM_2.5_	0.141	0.003	0.005	0.010	37.60	20
PM_2.5–10_	0.457	0.006	−0.005	0.899	1.62	5
PM_10_	0.289	0.005	−0.005	0.000	44.53	18
Nitrogen dioxide	0.659	0.003	0.001	0.004	58.33	33
Nitrogen oxides	0.189	0.004	0.005	0.000	64.13	29

GWAS, genome-wide association study; PM, Particulate matter; P, value of *p*; SE, standard error; Q, Q-value; Q-df, Q-degrees of freedom.

### 3.4. Sensitivity analysis

Sensitivity analysis of the MR results was performed. Leave-one-out sensitivity analysis for the impact of PM_2.5_ on frailty showed that all IV lines are on the right side of 0, and the results are unaffected significantly by the removal of any individual SNP (Figure 4). The fairly robust MR findings in this investigation indicate that PM_2.5_ is a risk factor for frailty. Additionally, analyses of other exposures revealed that eliminating each SNP individually had little impact on the outcomes, indicating that no one SNP had a major impact on the estimates of the overall causal effect (Figure 4).

## 4. Discussion

In this MR analysis, we examined the causal relationship between air pollution (including PM_2.5_, PM_2.5–10_, PM_10_, nitrogen dioxide and nitrogen oxides) and frailty. We found that increasing PM_2.5_ concentrations were associated with an increased risk of frailty in a European population in IVW method, with each standard deviation increase in PM_2.5_ being associated with a 33% increase in the risk of frailty by using a threshold of *p* < 1 × 10^−6^ for selection of instrumental variables (OR: 1.33; 95% CI: 1.12–1.58). The IVW method assumes that all genetic instruments are valid and that there is no horizontal pleiotropy (i.e., genetic variants only affect the frailty through the PM_2.5_). In addition, the weighted median approach which provides a causal estimate even when up to 50% of the genetic instruments are invalid also yielded significant results. However, the results of MR-Egger, weighted model and simple model tests were not significant. This result may be caused by residual pleiotropy. Furthermore, the results of the leave-one-out analysis and MR-Egger test were confirmed the robustness of this finding. In summary, our results suggest a strong causal relationship between PM_2.5_ concentration and frailty risk. No causal association was observed between other air pollutants (PM_2.5–10_, PM_10_, nitrogen dioxide and nitrogen oxides) and frailty.

The relationship between frailty and air pollution is an area of research that has received much attention. Frailty is a syndrome of old age which is largely influenced by a combination of environmental and genetic factors. Our findings further confirmed the conclusions of a number of epidemiologic studies that air pollutants are risk factors for frailty. For example, Eckel et al. examined the effect of a history of frailty on the correlation between PM_10_ and lung function using cohort data from adults aged ≥ 65 years in four US counties (36). In addition, a cross-sectional study reported that in Taiwan, frail adults aged ≥ 65 years had higher PM_2.5_ exposures than healthy participants (37). As mentioned previously, In the cross-sectional study of older adults in Korea, PM_2.5_, PM_10_, and ozone were commonly associated with frailty in a dose-dependent relationship with the degree of frailty (19). PM_2.5_ exposure was linked to an elevated risk of frailty in older persons living in rural areas in a sizable sample of community-based adults aged 50 and older from six middle-income nations (38). A nationwide prospective cohort study’s findings revealed a consistent link between Chinese older persons’ frailty and long-term PM_2.5_ exposure, and the significant decrease in PM_2.5_-related frailty burden was mainly due to the mitigation of PM_2.5_ (39). Similarly, a study by Guo et al. that included 13,910 participants aged ≥ 45 years showed that in middle-aged and older persons, long-term PM_2.5_ exposure was linked to a higher chance of frailty deterioration and a lower risk of improvement (20). Currently, research on the causal connection between air pollution and the risk of frailty is lacking. Therefore, we conducted a genetic-level causal analysis of air pollution and frailty using a two-sample Mendelian randomization method. Our findings showed a significant causal link between PM_2.5_ and the risk of frailty, which complements studies on the correlation between air pollutants and frailty.

PM_2.5_ has multiple effects on the risk of frailty in European populations, including respiratory problems, cardiovascular disease. These health problems may increase the occurrence of frailty, especially for people who already have health problems (40). PM_2.5_ particles are suspended in the air for long periods of time and are easily inhaled by the body. Once inhaled into the lungs, these tiny particles can cause a range of respiratory problems, including airway inflammation, bronchospasm and lung infections (41, 42). PM_2.5_ particulate matter has also been linked to the onset and progression of cardiovascular disease, increasing the risk of cardiovascular diseases such as heart disease, high blood pressure, and stroke (43–45). In Europe, cardiovascular disease is one of the major contributors to frailty (46).

The molecular mechanisms by which PM_2.5_ increases the risk of frailty are unknown. Evidence accumulated over the past two decades suggests that air pollution, along with consistent exposure to environment PM_2.5_, induces inflammation, persistent systemic oxidative stress, stimulation of the autonomic nervous system, and genetic and epigenetic alterations (47). PM_2.5_ poses an even greater health threat because of its small particle size, which enables it to cross several organ blood barriers, including those in the brain, liver, and kidneys, and reach the distal lungs, including the alveoli (48, 49). According to earlier research, elevated PM_2.5_ levels due to traffic are linked to different inflammatory marker responses (50). Additionally, it has been discovered that PM_2.5_ increases oxidative stress in the body, inhibits endogenous antioxidant enzyme activity and gene expression, and promotes dysfunction in a number of organs and systems (51–53). And factors such as inflammatory response and oxidative stress are believed to be pathological mechanisms leading to frailty (54). Studies have revealed that frail populations are more likely to experience low-grade inflammation, and that a high inflammatory state during middle age increases the possibility of developing frail in old age (55, 56). By encouraging the breakdown of muscle protein, inflammatory substances like C-reactive protein (CRP) and IL-6 with increasing quantities hasten the onset of frailty (57). Additionally, oxidative stress (OS) is also crucial in encouraging the emergence of frailty. Older persons with frailty have higher levels of OS and lower levels of antioxidants, according to a meta-analysis by Soysal et al. (58). The following are some potential processes by which OS encourages frailty: OS causes cellular damage, activates apoptosis pathways, increases transcription factor expression and protein degradation, and decreases mitochondrial function, and impairs repair mechanisms (59). Based on the facts mentioned above, we suspect that air pollutants (particularly PM_2.5_) cause the body to experience oxidative stress and inflammatory reactions, both of which result in cellular and molecular damage. The resulting damage exacerbates age-related decline and loss of functional characteristics at the cellular, tissue, and organ levels, which can result in cumulative decreases in a number of physiological systems, such as the immunological, endocrine, and skeletal muscle, and increase the prevalence of frailty.

Our MR study has a number of strengths. First, Mendel’s law of independent assignment chose genetic variation as the exposure factor when we utilized MR to investigate the causal link between air pollution and frailty, increasing the validity of the results. Second, reverse causation cannot exist because the genes appeared before the disease. Third, this study used data from the public GWAS pooled investigations, which has a considerable number of samples size and strengthens the test. In addition, the significance of any potential correlations resulting from population stratification is probably lessened because these people are of European heritage. This study bridges the gap between traditional observational studies and provides a fresh theoretical and experimental foundation for mitigating the threats that air pollutants offer to public health.

Inevitably, our research has some restrictions. First, further research on populations in other nations is required to increase the generalizability of the findings as the GWAS dataset used in the MR analyses is from Europe and this link may differ in people of other ancestries. Second, due to a paucity of SNPs associated with the 5 × 10^−8^ genome-wide significance threshold, our results were determined using a significance level of 1 × 10^−6^; nevertheless, extending the sample size may be necessary to further support our conclusions. Third, because we only employed summary statistics from the MR study, we were only able to make a preliminary determination about the causal link between PM_2.5_ and frailty. More research is still required to determine exactly how PM_2.5_ raises the risk of frailty.

## 5. Conclusion

Frailty is a dynamic process that worsens or improves over time, and its negative effects can be reversed, controlled, and prevented. Overall, our work offers genetic proof that exposure to high levels of PM_2.5_ raises the risk of frailty. This not only provides important evidence for the field of public health, but also emphasizes the potential harm of PM_2.5_ to the health of European populations. It would help policy makers and the public to better understand the urgency of air quality improvement. It also provides a basis for the government and environmental organizations to formulate more stringent air quality standards and policies to reduce PM_2.5_ pollution. Additionally, this study provides direction for future research to delve deeper into the biological mechanisms, potential confounders, and other environmental factors influencing the risk of frailty between PM_2.5_ and frailty. This will help to further understand and elucidate this causal relationship.

## Data availability statement

The datasets presented in this study can be found in online repositories. The names of the repository/repositories and accession number(s) can be found in the article/Supplementary material.

## Ethics statement

All datasets in the present study were downloaded from public databases (https://gwas.mrcieu.ac.uk/). These public databases allowed researchers to download and analyze public datasets for scientific purposes; thus, ethics approval was not required. The studies were conducted in accordance with the local legislation and institutional requirements. The participants provided their written informed consent to participate in this study.

## Author contributions

HaX: Conceptualization, Data curation, Formal analysis, Investigation, Methodology, Project administration, Software, Visualization, Writing – original draft, Writing – review & editing. SH: Conceptualization, Data curation, Formal analysis, Investigation, Methodology, Project administration, Software, Visualization, Writing – original draft, Writing – review & editing. WY: Data curation, Formal analysis, Investigation, Methodology, Software, Visualization, Writing – original draft, Writing – review & editing. WZ: Writing – original draft, Writing – review & editing. HuX: Writing – original draft, Writing – review & editing. SC: Conceptualization, Supervision, Writing – original draft, Writing – review & editing.
