# Supplementary material for: Causal association between air pollution and frailty: a Mendelian randomization study
Source: Front Public Health. 2023 Nov 7;11:1288293. doi: 10.3389/fpubh.2023.1288293 (PMC10662305; doi:10.3389/fpubh.2023.1288293)
Supplement: Supplementary file 1 [file Table_1.DOCX]

Supplementary Material

# Supplementary Table

Genetic variants significantly associated with air pollution exposure as instrumental variables and frailty as outcome.

| SNP | Effect allele | Other allele | Beta | SE | EAF | P | F | R^2^ |
| --- | --- | --- | --- | --- | --- | --- | --- | --- |
| **PM2.5:** | | | | | | | | |
| rs10172295 | A | G | -0.01133 | 0.002099 | 0.416147 | 6.80E-08 | 29.10969 | 6.38E-05 |
| rs11049241 | A | C | 0.013702 | 0.002654 | 0.186886 | 2.40E-07 | 26.64915 | 5.84E-05 |
| rs115860766 | T | C | 0.02001 | 0.003837 | 0.077984 | 1.80E-07 | 27.19749 | 5.96E-05 |
| rs11855821 | A | G | -0.01184 | 0.002336 | 0.282788 | 4.00E-07 | 25.6844 | 5.63E-05 |
| rs12089815 | A | G | -0.01062 | 0.002079 | 0.548637 | 3.30E-07 | 26.07579 | 5.71E-05 |
| rs1217106 | G | A | 0.014557 | 0.002519 | 0.782419 | 7.50E-09 | 33.40457 | 7.32E-05 |
| rs1318845 | C | T | -0.01417 | 0.002596 | 0.200795 | 4.80E-08 | 29.80215 | 6.53E-05 |
| rs13429081 | T | A | 0.013454 | 0.002661 | 0.186042 | 4.30E-07 | 25.55818 | 5.60E-05 |
| rs144720952 | G | A | 0.04988 | 0.009756 | 0.014107 | 3.20E-07 | 26.13908 | 5.73E-05 |
| rs17183854 | G | T | -0.04794 | 0.009091 | 0.013342 | 1.30E-07 | 27.81469 | 6.09E-05 |
| rs17513184 | T | C | -0.01994 | 0.003855 | 0.083064 | 2.30E-07 | 26.74638 | 5.86E-05 |
| rs27152 | T | C | -0.01187 | 0.002211 | 0.663321 | 8.00E-08 | 28.81832 | 6.31E-05 |
| rs329177 | A | G | 0.010877 | 0.002179 | 0.611738 | 6.00E-07 | 24.91621 | 5.46E-05 |
| rs34969378 | A | G | -0.01132 | 0.002225 | 0.316195 | 3.60E-07 | 25.88901 | 5.67E-05 |
| rs3740390 | T | C | 0.020166 | 0.003811 | 0.079749 | 1.20E-07 | 28.00148 | 6.14E-05 |
| rs4721275 | T | C | -0.02638 | 0.005381 | 0.961737 | 9.40E-07 | 24.03933 | 5.27E-05 |
| rs6667345 | T | C | 0.012721 | 0.002456 | 0.270243 | 2.20E-07 | 26.82426 | 5.88E-05 |
| rs6749467 | A | G | -0.01166 | 0.002096 | 0.46467 | 2.60E-08 | 30.95767 | 6.78E-05 |
| rs72642437 | T | C | 0.09054 | 0.018505 | 0.003807 | 9.90E-07 | 23.93825 | 5.24E-05 |
| rs72655898 | G | A | -0.01149 | 0.002302 | 0.286147 | 6.00E-07 | 24.91288 | 5.46E-05 |
| rs72808024 | C | A | -0.01703 | 0.002906 | 0.148435 | 4.60E-09 | 34.33557 | 7.52E-05 |
| rs73085844 | G | T | -0.03311 | 0.006535 | 0.025544 | 4.00E-07 | 25.6769 | 5.63E-05 |
| rs74957515 | G | C | 0.015755 | 0.003139 | 0.129158 | 5.20E-07 | 25.19622 | 5.52E-05 |
| rs7514956 | C | A | -0.01461 | 0.002652 | 0.186513 | 3.60E-08 | 30.34142 | 6.65E-05 |
| rs77205736 | T | C | 0.013265 | 0.002315 | 0.274855 | 1.00E-08 | 32.84414 | 7.20E-05 |
| rs77255816 | T | C | 0.029905 | 0.005471 | 0.036924 | 4.60E-08 | 29.87962 | 6.55E-05 |
| rs7776279 | A | G | 0.01165 | 0.002292 | 0.714735 | 3.70E-07 | 25.83007 | 5.66E-05 |
| rs7910200 | T | C | -0.01042 | 0.002089 | 0.424276 | 6.10E-07 | 24.87442 | 5.45E-05 |
| rs8614 | A | C | 0.013909 | 0.002677 | 0.182486 | 2.00E-07 | 26.99597 | 5.91E-05 |
| rs9495443 | C | A | -0.01098 | 0.002215 | 0.334297 | 7.10E-07 | 24.59202 | 5.39E-05 |
| **PM2.5-10:** | | | | | | | | |
| rs1157546 | C | T | -0.0246 | 0.004901 | 0.053047 | 5.20E-07 | 25.20466 | 5.95E-05 |
| rs11621531 | A | G | -0.02313 | 0.004487 | 0.063928 | 2.50E-07 | 26.56463 | 6.27E-05 |
| rs12462492 | T | G | -0.01239 | 0.002496 | 0.257064 | 6.90E-07 | 24.64163 | 5.81E-05 |
| rs1706918 | A | G | 0.018349 | 0.003677 | 0.098932 | 6.00E-07 | 24.90627 | 5.88E-05 |
| rs78060907 | A | C | -0.03805 | 0.007698 | 0.020337 | 7.70E-07 | 24.43938 | 5.77E-05 |
| rs9497937 | A | C | -0.01277 | 0.002577 | 0.232437 | 7.20E-07 | 24.55547 | 5.79E-05 |
| **PM10:** | | | | | | | | |
| rs10498638 | C | T | 0.014014 | 0.002537 | 0.188322 | 3.30E-08 | 30.51274 | 6.70E-05 |
| rs13084230 | T | C | -0.01356 | 0.00246 | 0.200319 | 3.50E-08 | 30.3951 | 6.68E-05 |
| rs13122455 | T | C | -0.014 | 0.002464 | 0.199995 | 1.30E-08 | 32.29304 | 7.09E-05 |
| rs140295641 | A | T | -0.03514 | 0.006172 | 0.027357 | 1.30E-08 | 32.4059 | 7.12E-05 |
| rs142169179 | A | G | 0.040185 | 0.00734 | 0.020279 | 4.40E-08 | 29.97081 | 6.58E-05 |
| rs147895162 | C | T | -0.04476 | 0.008107 | 0.015135 | 3.40E-08 | 30.48057 | 6.69E-05 |
| rs2004679 | C | T | 0.011915 | 0.002138 | 0.307685 | 2.50E-08 | 31.0491 | 6.82E-05 |
| rs2248162 | C | T | 0.011819 | 0.002047 | 0.63988 | 7.80E-09 | 33.33063 | 7.32E-05 |
| rs4788565 | A | G | -0.02192 | 0.003994 | 0.066776 | 4.10E-08 | 30.1107 | 6.61E-05 |
| rs4833095 | C | T | 0.025111 | 0.002406 | 0.206945 | 1.70E-25 | 108.9642 | 0.000239 |
| rs56084453 | G | A | 0.014935 | 0.002412 | 0.209746 | 5.90E-10 | 38.35161 | 8.42E-05 |
| rs60304336 | T | G | 0.027932 | 0.00503 | 0.040906 | 2.80E-08 | 30.84178 | 6.77E-05 |
| rs61620752 | G | T | 0.016069 | 0.002767 | 0.148343 | 6.40E-09 | 33.71954 | 7.41E-05 |
| rs61875074 | C | A | 0.022266 | 0.003841 | 0.073029 | 6.80E-09 | 33.60045 | 7.38E-05 |
| rs6793835 | A | G | -0.01299 | 0.002238 | 0.263796 | 6.60E-09 | 33.66093 | 7.39E-05 |
| rs6867849 | T | A | -0.03148 | 0.005232 | 0.040477 | 1.80E-09 | 36.19834 | 7.95E-05 |
| rs7200852 | A | C | -0.02443 | 0.00445 | 0.054732 | 4.00E-08 | 30.12926 | 6.62E-05 |
| rs74247887 | T | C | 0.037132 | 0.005881 | 0.028721 | 2.70E-10 | 39.86967 | 8.76E-05 |
| rs9640029 | T | C | -0.0138 | 0.001974 | 0.478233 | 2.70E-12 | 48.89763 | 0.000107 |
| **Nitrogen dioxide:** | | | | | | | | |
| rs10498638 | C | T | 0.01284 | 0.00261 | 0.188308 | 8.70E-07 | 24.20714 | 5.30E-05 |
| rs10804931 | T | G | -0.01031 | 0.002097 | 0.622587 | 8.70E-07 | 24.18812 | 5.30E-05 |
| rs10983735 | A | G | 0.016311 | 0.002803 | 0.154461 | 5.90E-09 | 33.86667 | 7.42E-05 |
| rs11191447 | T | C | 0.020186 | 0.003733 | 0.079724 | 6.40E-08 | 29.23217 | 6.40E-05 |
| rs112429058 | G | A | 0.026038 | 0.005294 | 0.038211 | 8.70E-07 | 24.19035 | 5.30E-05 |
| rs11609798 | C | G | -0.01017 | 0.002064 | 0.41241 | 8.40E-07 | 24.27121 | 5.32E-05 |
| rs116289821 | A | G | -0.03378 | 0.006864 | 0.022548 | 8.60E-07 | 24.21051 | 5.30E-05 |
| rs149827261 | T | G | -0.01501 | 0.00292 | 0.146904 | 2.70E-07 | 26.43029 | 5.79E-05 |
| rs1502243 | A | G | -0.01172 | 0.00237 | 0.241027 | 7.70E-07 | 24.43644 | 5.35E-05 |
| rs1978728 | T | C | -0.01102 | 0.002108 | 0.360516 | 1.70E-07 | 27.32747 | 5.99E-05 |
| rs2290154 | C | T | 0.011101 | 0.002116 | 0.358312 | 1.50E-07 | 27.53594 | 6.03E-05 |
| rs2301747 | G | C | 0.015367 | 0.003082 | 0.121164 | 6.10E-07 | 24.86476 | 5.45E-05 |
| rs2517897 | A | C | 0.014476 | 0.002814 | 0.152648 | 2.70E-07 | 26.45788 | 5.80E-05 |
| rs2926098 | T | C | -0.01907 | 0.003709 | 0.081135 | 2.70E-07 | 26.45231 | 5.80E-05 |
| rs34623735 | T | C | 0.01272 | 0.002157 | 0.33449 | 3.70E-09 | 34.77465 | 7.62E-05 |
| rs35544454 | T | A | -0.01296 | 0.002635 | 0.181817 | 8.80E-07 | 24.18297 | 5.30E-05 |
| rs35706972 | A | G | 0.043284 | 0.008744 | 0.014251 | 7.40E-07 | 24.50291 | 5.37E-05 |
| rs35731545 | T | C | -0.03921 | 0.007774 | 0.018156 | 4.60E-07 | 25.43806 | 5.57E-05 |
| rs4105558 | G | C | -0.01144 | 0.002181 | 0.314508 | 1.60E-07 | 27.50874 | 6.03E-05 |
| rs55643000 | G | T | -0.02125 | 0.004237 | 0.062116 | 5.30E-07 | 25.15956 | 5.51E-05 |
| rs62062033 | T | C | 0.014352 | 0.002719 | 0.172431 | 1.30E-07 | 27.85629 | 6.10E-05 |
| rs62180536 | T | C | -0.0321 | 0.006078 | 0.028932 | 1.30E-07 | 27.88851 | 6.11E-05 |
| rs62458757 | G | C | -0.02912 | 0.005628 | 0.034938 | 2.30E-07 | 26.76558 | 5.86E-05 |
| rs6658422 | T | C | 0.024908 | 0.005055 | 0.041642 | 8.30E-07 | 24.28291 | 5.32E-05 |
| rs6954825 | C | T | 0.014131 | 0.002765 | 0.158973 | 3.20E-07 | 26.12111 | 5.72E-05 |
| rs7225402 | C | T | -0.02489 | 0.004315 | 0.058308 | 8.00E-09 | 33.27302 | 7.29E-05 |
| rs72642437 | T | C | 0.091111 | 0.018141 | 0.003807 | 5.10E-07 | 25.22325 | 5.53E-05 |
| rs72839513 | A | G | 0.020894 | 0.004186 | 0.062553 | 6.00E-07 | 24.9098 | 5.46E-05 |
| rs73072692 | T | C | 0.012482 | 0.002413 | 0.236944 | 2.30E-07 | 26.75128 | 5.86E-05 |
| rs73178577 | A | C | 0.018359 | 0.003691 | 0.082709 | 6.50E-07 | 24.74664 | 5.42E-05 |
| rs77205736 | T | C | 0.015406 | 0.002269 | 0.274855 | 1.10E-11 | 46.11058 | 0.000101 |
| rs79475047 | C | T | 0.032731 | 0.006197 | 0.027316 | 1.30E-07 | 27.8928 | 6.11E-05 |
| rs9541967 | C | T | -0.01611 | 0.003077 | 0.123504 | 1.60E-07 | 27.41337 | 6.01E-05 |
| rs974801 | G | A | 0.010699 | 0.002102 | 0.364613 | 3.60E-07 | 25.90711 | 5.68E-05 |
| **Nitrogen oxides:** | | | | | | | | |
| rs10172295 | A | G | -0.01133 | 0.002099 | 0.416147 | 6.80E-08 | 29.10969 | 6.38E-05 |
| rs11049241 | A | C | 0.013702 | 0.002654 | 0.186886 | 2.40E-07 | 26.64915 | 5.84E-05 |
| rs115860766 | T | C | 0.02001 | 0.003837 | 0.077984 | 1.80E-07 | 27.19749 | 5.96E-05 |
| rs11855821 | A | G | -0.01184 | 0.002336 | 0.282788 | 4.00E-07 | 25.6844 | 5.63E-05 |
| rs12089815 | A | G | -0.01062 | 0.002079 | 0.548637 | 3.30E-07 | 26.07579 | 5.71E-05 |
| rs1217106 | G | A | 0.014557 | 0.002519 | 0.782419 | 7.50E-09 | 33.40457 | 7.32E-05 |
| rs1318845 | C | T | -0.01417 | 0.002596 | 0.200795 | 4.80E-08 | 29.80215 | 6.53E-05 |
| rs13429081 | T | A | 0.013454 | 0.002661 | 0.186042 | 4.30E-07 | 25.55818 | 5.60E-05 |
| rs144720952 | G | A | 0.04988 | 0.009756 | 0.014107 | 3.20E-07 | 26.13908 | 5.73E-05 |
| rs17183854 | G | T | -0.04794 | 0.009091 | 0.013342 | 1.30E-07 | 27.81469 | 6.09E-05 |
| rs17513184 | T | C | -0.01994 | 0.003855 | 0.083064 | 2.30E-07 | 26.74638 | 5.86E-05 |
| rs27152 | T | C | -0.01187 | 0.002211 | 0.663321 | 8.00E-08 | 28.81832 | 6.31E-05 |
| rs329177 | A | G | 0.010877 | 0.002179 | 0.611738 | 6.00E-07 | 24.91621 | 5.46E-05 |
| rs34969378 | A | G | -0.01132 | 0.002225 | 0.316195 | 3.60E-07 | 25.88901 | 5.67E-05 |
| rs3740390 | T | C | 0.020166 | 0.003811 | 0.079749 | 1.20E-07 | 28.00148 | 6.14E-05 |
| rs4721275 | T | C | -0.02638 | 0.005381 | 0.961737 | 9.40E-07 | 24.03933 | 5.27E-05 |
| rs6667345 | T | C | 0.012721 | 0.002456 | 0.270243 | 2.20E-07 | 26.82426 | 5.88E-05 |
| rs6749467 | A | G | -0.01166 | 0.002096 | 0.46467 | 2.60E-08 | 30.95767 | 6.78E-05 |
| rs72642437 | T | C | 0.09054 | 0.018505 | 0.003807 | 9.90E-07 | 23.93825 | 5.24E-05 |
| rs72655898 | G | A | -0.01149 | 0.002302 | 0.286147 | 6.00E-07 | 24.91288 | 5.46E-05 |
| rs72808024 | C | A | -0.01703 | 0.002906 | 0.148435 | 4.60E-09 | 34.33557 | 7.52E-05 |
| rs73085844 | G | T | -0.03311 | 0.006535 | 0.025544 | 4.00E-07 | 25.6769 | 5.63E-05 |
| rs74957515 | G | C | 0.015755 | 0.003139 | 0.129158 | 5.20E-07 | 25.19622 | 5.52E-05 |
| rs7514956 | C | A | -0.01461 | 0.002652 | 0.186513 | 3.60E-08 | 30.34142 | 6.65E-05 |
| rs77205736 | T | C | 0.013265 | 0.002315 | 0.274855 | 1.00E-08 | 32.84414 | 7.20E-05 |
| rs77255816 | T | C | 0.029905 | 0.005471 | 0.036924 | 4.60E-08 | 29.87962 | 6.55E-05 |
| rs7776279 | A | G | 0.01165 | 0.002292 | 0.714735 | 3.70E-07 | 25.83007 | 5.66E-05 |
| rs7910200 | T | C | -0.01042 | 0.002089 | 0.424276 | 6.10E-07 | 24.87442 | 5.45E-05 |
| rs8614 | A | C | 0.013909 | 0.002677 | 0.182486 | 2.00E-07 | 26.99597 | 5.91E-05 |
| rs9495443 | C | A | -0.01098 | 0.002215 | 0.334297 | 7.10E-07 | 24.59202 | 5.39E-05 |
